# Large chemokine binding spectrum of human and mouse atypical chemokine receptor GPR182 (ACKR5)

**DOI:** 10.3389/fphar.2023.1297596

**Published:** 2023-11-01

**Authors:** Remy Bonnavion, Shangmin Liu, Haruya Kawase, Kenneth Anthony Roquid, Stefan Offermanns

**Affiliations:** ^1^ Department of Pharmacology, Max Planck Institute for Heart and Lung Research, Bad Nauheim, Germany; ^2^ Cardiopulmonary Institute, Bad Nauheim, Germany; ^3^ Center for Molecular Medicine, Goethe University Frankfurt, Frankfurt, Germany; ^4^ German Center for Cardiovascular Research, Partner Site Frankfurt, Bad Nauheim, Germany

**Keywords:** GPCR, ACKR, chemokine, scavenger, receptor binding

## Abstract

Atypical chemokine receptors (ACKRs) play pivotal roles in immune regulation by binding chemokines and regulating their spatial distribution without inducing G-protein activation. Recently, GPR182, provisionally named ACKR5, was identified as a novel ACKR expressed in microvascular and lymphatic endothelial cells, with functions in hematopoietic stem cell homeostasis. Here, we comprehensively investigated the chemokine binding profile of human and mouse GPR182. Competitive binding assays using flow cytometry revealed that besides CXCL10, CXCL12 and CXCL13, also human and mouse CXCL11, CXCL14 and CCL25, as well as human CCL1, CCL11, CCL19, CCL26, XCL1 and mouse CCL22, CCL24, CCL27 and CCL28 bind with an affinity of less than 100 nM to GPR182. In line with the binding affinity observed *in vitro*, elevated serum levels of CCL22, CCL24, CCL25, and CCL27 were observed in GPR182-deficient mice, underscoring the role of GPR182 in chemokine scavenging. These data show a broader chemokine binding repertoire of GPR182 than previously reported and they will be important for future work exploring the physiological and pathophysiological roles of GPR182, which we propose to be renamed atypical chemokine receptor 5 (ACKR5).

## Introduction

Atypical chemokine receptors (ACKRs) bind chemokines but do not lead to G-protein activation and subsequent cellular effects. Instead, they recruit β-arrestin, which promotes chemokine uptake and scavenging to remove chemokines from the extracellular space and to limit the spatial availability of the chemokines (Bonecchi and Graham, 2016). There are 4 established ACKRs, ACKR1-4 (Bonecchi and Graham, 2016), and recently GPR182 has been discovered as a novel ACKR (Le Mercier et al., 2021), provisionally named ACKR5. GPR182 is expressed in microvascular and lymphatic endothelial cells (Xiao et al., 2014; Schmid et al., 2018; Le Mercier et al., 2021) and is involved in hematopoietic stem cell homeostasis (Le Mercier et al., 2021). Similar to other ACKRs, it does not couple to G-proteins, but also does not show ligand-dependent recruitment of β-arrestin (Le Mercier et al., 2021). GPR182 has instead high constitutive activity to recruit β-arrestin and therefore rapidly internalizes in a ligand-independent manner (Le Mercier et al., 2021). These properties have been confirmed by an independent study (Torphy et al., 2022), which provided evidence for a role of lymphatic endothelial GPR182 in limiting anti-tumor immunity. Both studies, however, differed with regard to the described spectrum of chemokines binding to GPR182. Le Mercier et al. reported that GPR182 is a high-affinity ACKR for CXCL10, CXCL12, and CXCL13, with affinities in the range of 10–50 nM and that plasma levels of CXCL10, CXCL12 and CXCL13 were elevated in GPR182-deficient mice (Le Mercier et al., 2021). In contrast, Torphy et al. identified CXCL9 as a low-affinity ligand for GPR182 with an affinity of 1.33 μM, and in competition binding assays showed that various other chemokines, given at a concentration of 2 µM were able to compete with the low-affinity GPR182 ligand CXCL9 (Torphy et al., 2022). In order to reconcile both studies, we comprehensively investigated the chemokine binding profile of human and mouse GPR182 using competition binding assays in cells expressing human or mouse GPR182.

## Methods

### For extended methods: see supplement

HEK-293 cells stably expressing human GPR182 or mouse GPR182 were generated by lentiviral transduction followed by puromycin selection.

Equilibrium binding of AlexaFluor-647 labelled human CXCL10 (CXCL10-AF647: CAF-10, Almac) to human and mouse GPR182 was performed in single cell suspensions obtained by trypsin/EDTA treatment of cultured parental HEK-293 cells or HEK-293 cells stably expressing human or mouse GPR182. Cells were resuspended in cold (4 °C) binding buffer (125 mM NaCl, 5.9 mM KCl, 1 mM MgCl_2_, 2.56 mM CaCl_2_, 25 mM Hepes (pH 7.4)). CXCL10-AF647 was added to the cells and cells were then incubated for 1 h under gentle shaking at 4°C to avoid receptor internalization. Thereafter, cells were washed twice with binding buffer and fixed for 10 min with 1% PFA diluted in binding buffer on ice. After centrifugation, cells were resuspended in binding buffer and the binding of CXCL10-AF647 to the cells was determined by flow cytometry (BD FACS Canto II) after addition of DAPI to exclude dead cells.

Competition binding assays were performed at 4 °C by incubating non-labeled recombinant chemokine from Peprotech or Biolegend shortly before adding fluorescently labelled CXCL10-AF647.

Serum from wild-type or GPR182^−/−^ mice (Le Mercier et al., 2021) were prepared and used to determine chemokine levels using specific ELISA kits following manufacturer’s instructions.

## Results

**FIGURE 1:**
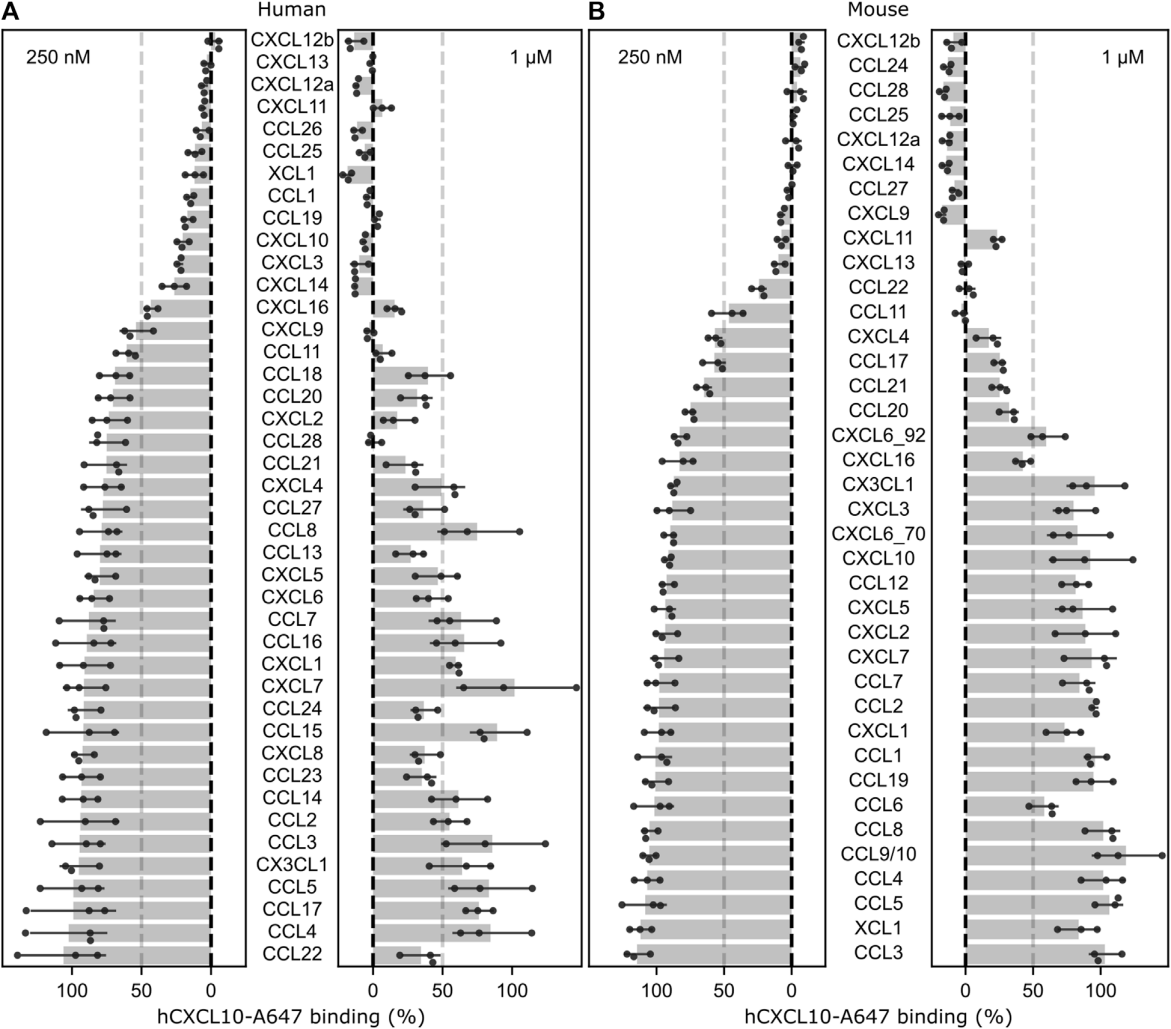
Screen of chemokine binding to human and mouse GPR182. Effect of human **(A)** or mouse **(B)** chemokines at 250 nM or 1 µM on the binding of 50 nM of human CXCL10-A647 to human or murine GPR182. The dotted-line represents 50% displacement of labelled human CXCL10. Shown are mean values ± S.D (n = 3 biological replicates; shown is a representative of 2 independently performed experiments).

To describe the GPR182 chemokine binding specificity, we have systematically analyzed the binding of 42 human and 38 murine chemokines to human and mouse GPR182 in competitive binding assays at a low (250 nM) and a high (1 µM) ligand concentration using AlexaFluor-647 labelled human CXCL10 at a concentration of 50 nM (Figures 1A, B). Human CXCL10-AF647 had been shown to bind to the human and mouse GPR182 with a K_D_ of 128 and 330 nM, respectively (Supplementary Figures S1A and S1B) (Le Mercier et al., 2021). The results confirmed the ability of CXCL10, CXCL12 and CXCL13 to bind at low concentration to GPR182 but revealed that also other chemokines are able to displace CXCL10 from GPR182 at relatively low concentration, including human and mouse CXCL9, CXCL11, CXCL14, CCL11, CCL25, CCL28, human CXCL3, CXCL16, CCL1, CCL19, CCL26, XCL1 and mouse CCL22, CCL24, and CCL27 (Figures 1A, B).

**FIGURE 2:**
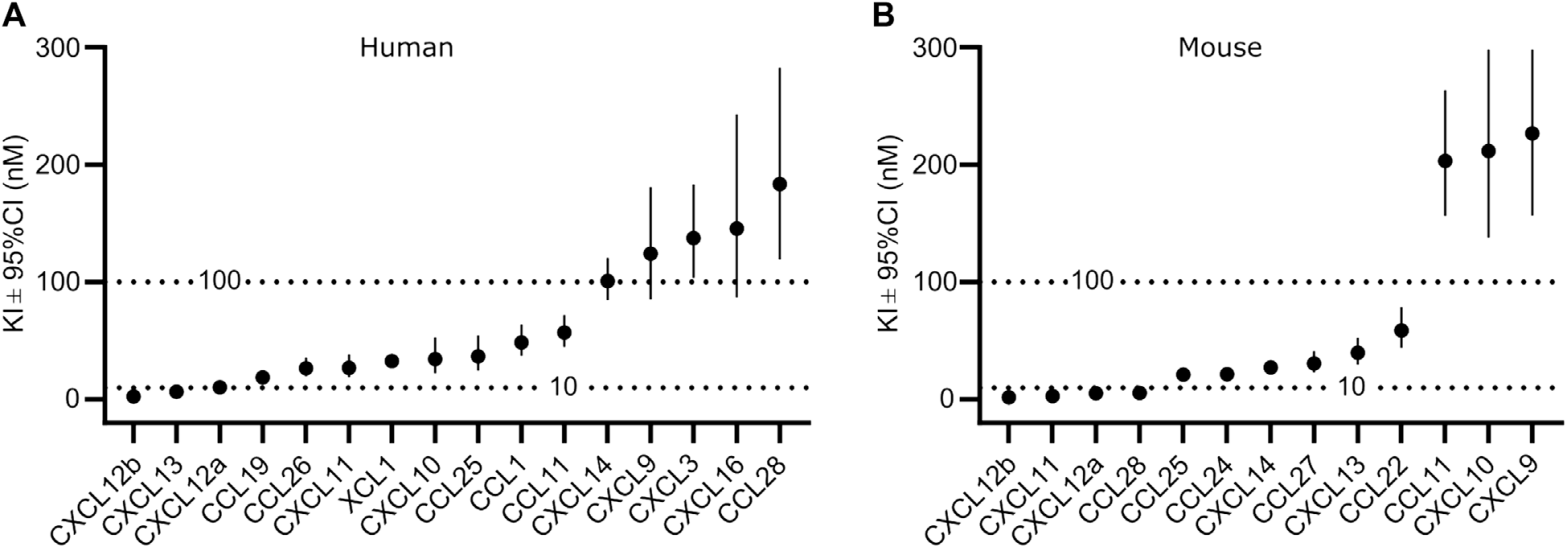
Affinity of chemokine binding to human and mouse GPR182. K_i_ values of the binding of human **(A)** or mouse **(B)** chemokines to the human and mouse GPR182 receptor, respectively as shown in Supplementary Figures S2 and S3. Shown are the calculated K_i_ values ± upper and lower limit of 95% confidence interval (Shown is a representative of 2 independently performed experiments).

To analyze the binding in more detail, we determined K_i_ values by competitive binding assays of those chemokines, which displaced at least 80% of CXCL10 at a concentration of 1 µM (Supplementary Figures S2, S3). A total of 16 human and 13 murine chemokines bound to the respective GPR182 with K_i_ values of ≤300 nM (Figures 2A, B and Supplementary Figures S2, S3). Among the high-affinity binding group (K_i_ ≤ 10 nM) were human CXCL12a, CXCL12b and CXCL13 as well as mouse CXCL12a, CXCL12b, CXCL11 and CCL28 (Figures 2A, B). In the group of chemokines with intermediate affinity (K_i_ = 10–100 nM) were human CXCL10, CXCL11, CCL1, CCL11, CCL19, CCL25, CCL26, XCL1 and mouse CXCL13, CXCL14, CCL22, CCL24, CCL25 and CCL27. In the group of low-affinity binding chemokines (K_i_ = 100–300 nM) were human CXCL3, CXCL9, CXCL14, CXCL16, CCL28 and mouse CXCL9, CXCL10 and CCL11 (Figures 2A, B and Supplementary Figures S2 and S3).

**FIGURE 3:**
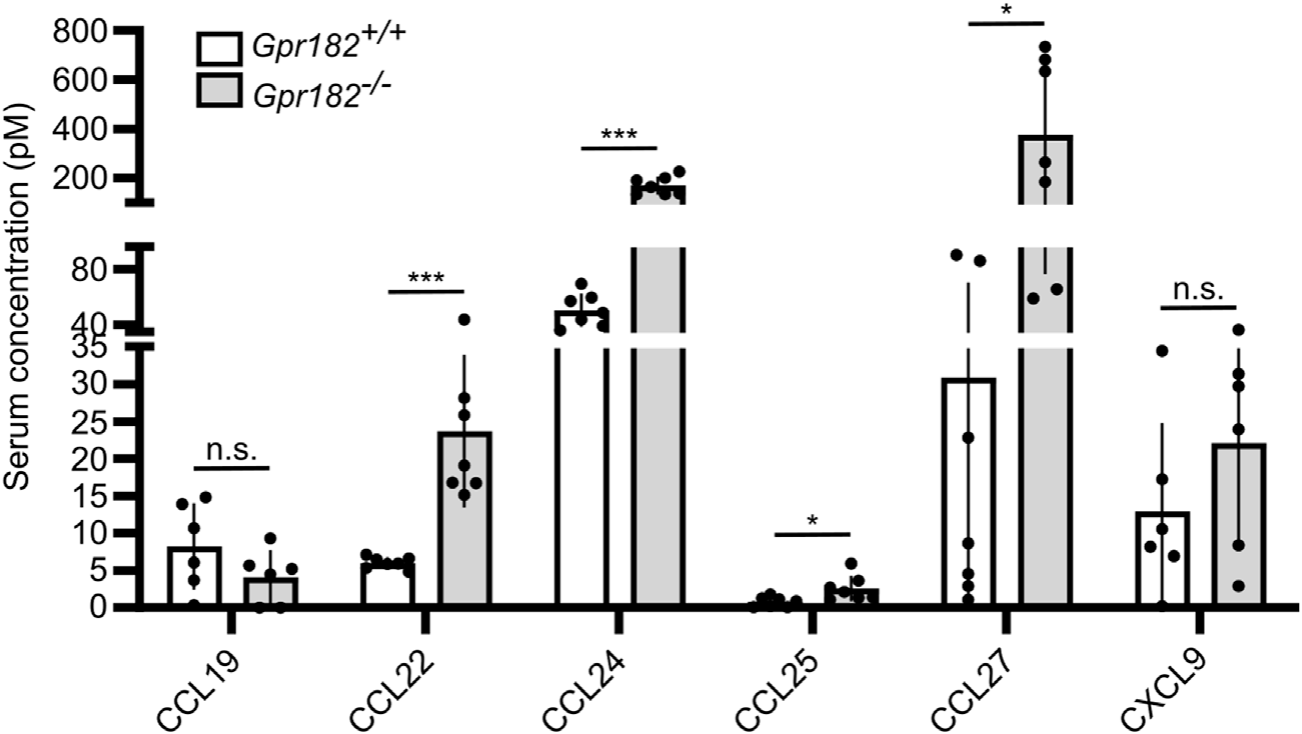
Chemokines levels in GPR182 deficient mice. Concentration of chemokines in the serum of *Gpr182* ^*−/−*^ mice and littermate controls (n = 7 mice per group or 6 mice per group (CCL19 and CXCL9); Shown are mean values ± S.D.; *, *p* ≤ 0.05; ***, *p* ≤ 0.001; n.s., non significant (unpaired two-tailed Student’s t-test).

Since it had previously been shown that high-affinity GPR182 ligands, such as CXCL10, CXCL12 and CXCL13, show increased serum levels in GPR182-deficient mice (Le Mercier et al., 2021), we determined levels of several chemokines binding to GPR182 with high or intermediate affinity (K_i_ ≤ 100 nM). We found that, in addition to CXCL10, CCL12 and CXCL13 (Le Mercier et al., 2021), also serum levels of CCL22, CCL24, CCL25 and CCL27 were increased in the absence of GPR182 (Figure 3), whereas levels of CCL19, which does not bind mouse GPR182, and CXCL9, which binds mouse GPR182 with low affinity, were unchanged (Figure 3). This supports the concept that GPR182 functions as a scavenger receptor of various chemokines to remove them from the extracellular space.

## Discussion

**FIGURE 4:**
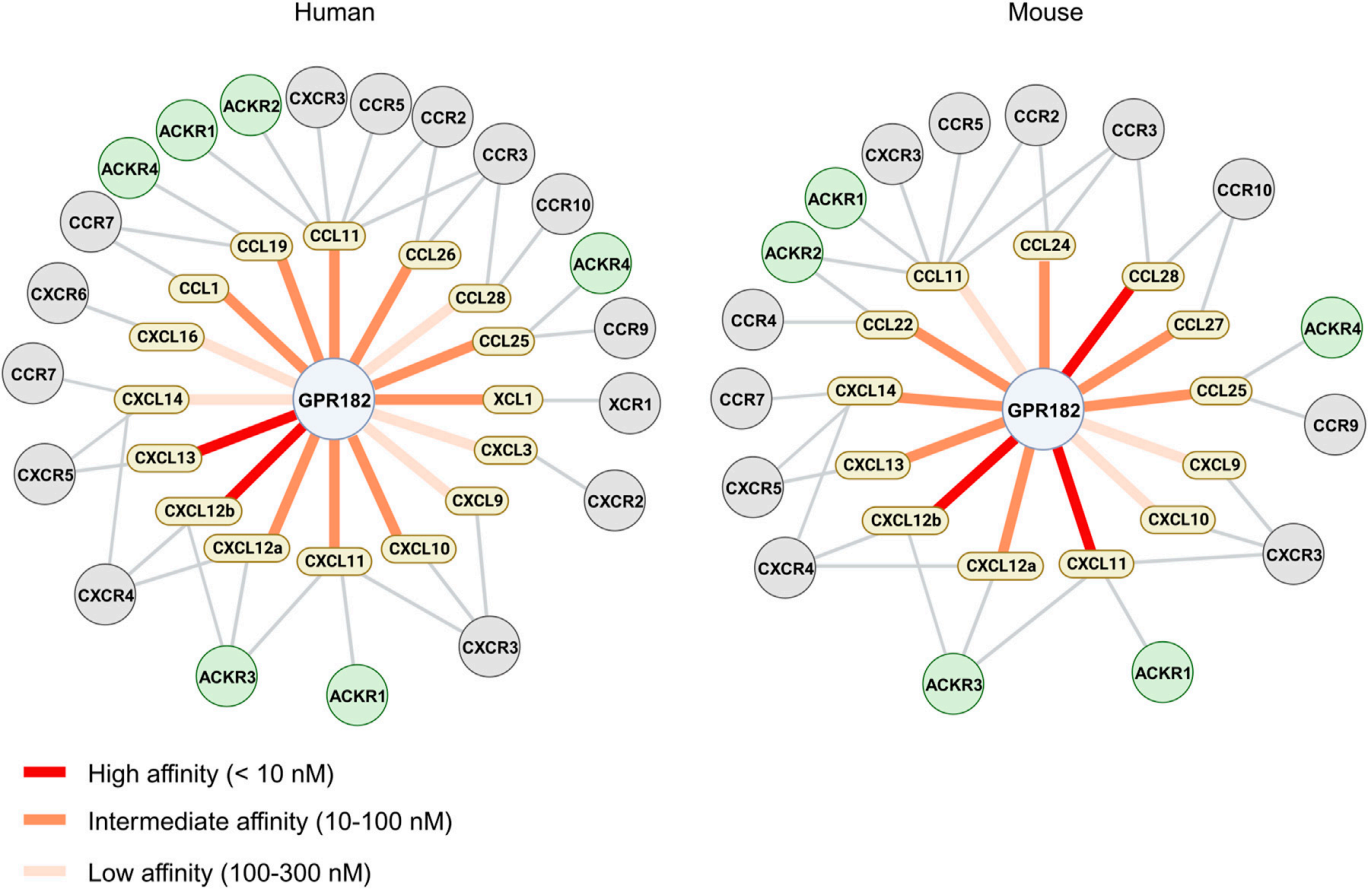
Chemokine binding pattern of GPR182. Schematic representation of the chemokine binding pattern of human (left) and mouse (right) GPR182 including other chemokine receptors which have been described to bind chemokines which interact with GPR182.

In this study we systematically evaluated the spectrum of chemokines binding to human and mouse GPR182. We confirmed high affinity binding of CXCL10, 12 and 13. In addition, we identified several other chemokines which bind to GPR182 with high, intermediate or low affinity and in some cases with species-specific differences (Figure 4). This considerably wider spectrum of GPR182 chemokine ligands was not observed in our initial study of chemokine binding to GPR182 (Le Mercier et al., 2021). One likely reason is the use of much higher chemokine concentrations in the competition binding assays of the present study which allow for identification of ligands with intermediate and low affinity. Also, receptor binding experiments were performed now at 4°C and not at room temperature to avoid confounding effects due to receptor internalization and recycling. In addition, instead of epifluorescence microscopy, we now used flow cytometry to determine GPR182 binding, which is a much more robust and precise way to quantify binding.

Our data confirm that human CXCL9 is a low-affinity ligand of GPR182 (Torphy et al., 2022). During the preparation of this manuscript, Melgrati et al. reported on a study which analyzed the binding of a subset of 10 chemokines to GPR182 (Melgrati et al., 2023). Their data indicate that mouse CCL19, CCL20 and CCL28 as well as human CCL19, CXCL12 and CXCL13 bind at 4°C to their respective GPR182 receptor. They also observed GPR182-dependent internalization of mouse CXCL13, CCL2, CCL28, CCL19, CCL20, CCL21, CCL25 and of human CXCL11, CXCL12, CXCL13, CCL19, CCL20 and CCL21 at 37°C. With regard to the chemokines analyzed by Melgrati et al., our results only differ for mouse CCL2 and CCL19 for which we did not observe binding even at 1 µM (Figure 1). The source of chemokine and cell lines used for binding and internalization experiments may be responsible for these differences.

Among the chemokines not tested by Melgrati et al., we found several additional mouse and human chemokines which bind to their respective GPR182 receptor with intermediate (10–100 nM) or low affinity (100–300 nM) (Figures 2, 4) Our data also indicate that human CCL28 has a much lower affinity for human GPR182 than mouse CCL28 to mouse GPR182. In addition to CCL28, we also observed species-specific differences in chemokine binding between the mouse and human systems. In contrast to the human system, mouse XCL1, CCL1, CCL19, CXCL3 and CXCL16 did not bind to mouse GPR182 (Figures 1A, B).

We also determined in GPR182 deficient mice the serum levels of several chemokines that we have identified to bind mouse GPR182. We found that not only serum levels of CXCL10, CXCL12 and CXCL13 (Le Mercier et al., 2021), but also those of CCL22, CCL24, CCL25 and CCL27 are significantly increased in the absence of GPR182, which underscores the scavenging function of GPR182. Interestingly, CCL19 serum levels were not dysregulated in GPR182 deficient mice, supporting our *in vitro* results that mouse CCL19 does not bind mouse GPR182.

In conclusion, these data define the spectrum of chemokines interacting with the atypical chemokine receptor GPR182 and show that GPR182 binds more than a third of the human and murine chemokines with an affinity between 2 and 300 nM and with some species specificities (Figure 4). Future work will further explore the physiological and pathophysiological function of GPR182, which we propose to be renamed atypical chemokine receptor 5 (ACKR5).

## Data Availability

The raw data supporting the conclusion of this article will be made available by the authors, without undue reservation.

## References

[B1] Bonecchi R. Graham G. J. (2016). Atypical chemokine receptors and their roles in the resolution of the inflammatory response. Front. Immunol. 7, 224. 10.3389/fimmu.2016.00224 27375622 PMC4901034

[B2] Le Mercier A. Bonnavion R. Yu W. Alnouri M. W. Ramas S. Zhang Y. (2021). GPR182 is an endothelium-specific atypical chemokine receptor that maintains hematopoietic stem cell homeostasis. Proc. Natl. Acad. Sci. U. S. A. 118, e2021596118. 10.1073/pnas.2021596118 33875597 PMC8092405

[B3] Melgrati S. Gerken O. J. Artinger M. Radice E. Szpakowska M. Chevigne A. (2023). GPR182 is a broadly scavenging atypical chemokine receptor influencing T-independent immunity. Front. Immunol. 14, 1242531. 10.3389/fimmu.2023.1242531 37554323 PMC10405735

[B4] Schmid C. D. Schledzewski K. Mogler C. Waldburger N. Kalna V. Marx A. (2018). GPR182 is a novel marker for sinusoidal endothelial differentiation with distinct GPCR signaling activity *in vitro* . Biochem. Biophys. Res. Commun. 497, 32–38. 10.1016/j.bbrc.2018.01.185 29408502

[B5] Torphy R. J. Sun Y. Lin R. Caffrey-Carr A. Fujiwara Y. Ho F. (2022). GPR182 limits antitumor immunity via chemokine scavenging in mouse melanoma models. Nat. Commun. 13, 97. 10.1038/s41467-021-27658-x 35013216 PMC8748779

[B6] Xiao L. Harrell J. C. Perou C. M. Dudley A. C. (2014). Identification of a stable molecular signature in mammary tumor endothelial cells that persists *in vitro* . Angiogenesis 17, 511–518. 10.1007/s10456-013-9409-y 24257808 PMC4029871

