# Supplementary material for: Large chemokine binding spectrum of human and mouse atypical chemokine receptor GPR182 (ACKR5)
Source: Front Pharmacol. 2023 Nov 1;14:1297596. doi: 10.3389/fphar.2023.1297596 (PMC10646305; doi:10.3389/fphar.2023.1297596)
Supplement: Supplementary file 1 [file DataSheet1.pdf]

**Supplemental Material**

# Characterization of the Chemokine Binding Spectrum of Human and Mouse Atypical Chemokine Receptor GPR182 (ACKR5)

Remy Bonnavion, Shangmin Liu, Haruya Kawase, Kenneth Anthony Roquid, Stefan Offermanns

## Supplemental Methods

### Cell Lines

The parental HEK-293 cells were obtained from the German Collection of Microorganisms and Cell Cultures GmbH (DSMZ; no.: ACC 305). HEK-293 cells stably expressing human GPR182 or mouse GPR182 were generated by lentiviral transduction followed by puromycin selection. The lentivirus used were carrying the sequence of a signal peptide from hemagglutinin followed by the coding sequence of either mouse or human GPR182 under the control of a CMV promoter. Multiclonal lines were derived by sorting cells with high GPR182 expression by flow cytometry using an antibody specific for GPR182 (see list of reagents). All cell lines were maintained in Dulbecco's modified Eagle's medium (DMEM) supplemented with 10% FBS, 2 mM L-glutamine, 5 units/ml penicillin/streptomycin.

### Ligand Binding Assay

Equilibrium binding of AlexaFluor-647 labelled human CXCL10 (CXCL10-AF647: CAF-10, Almac) to human and mouse GPR182 was performed in single cell suspensions of parental HEK-293 cells or HEK-293 cells stably expressing human or mouse GPR182. Single-cell suspensions were prepared by treatment of cells with 2.5 g/ml trypsin and 0.9 mM

ethylenediaminetetraacetic acid (EDTA) for 3 min at 37 °C. After inactivation using complete medium (see above) and centrifugation (300 x g for 5 min at room temperature), cells were resuspended in cold (4 °C) binding buffer (125 mM NaCl, 5.9 mM KCl, 1 mM $MgCl_2$, 2.56 mM $CaCl_2$, 25 mM Hepes (pH 7.4)), and 5 x $10^4$ cells in 40 µl were distributed per well on a V-bottom polypropylene 96 well-plate. CXCL10-AF647 was added to the cells at different concentrations to a final volume of 50 µl, and cells were then incubated for 1h under gentle shaking at 4°C to avoid receptor internalization. Thereafter, cells were washed twice with binding buffer and fixed for 10 min with 1% PFA diluted in binding buffer on ice. After centrifugation to remove PFA (350 x g for 6 min at 4 °C), cells were resuspended in 200 µl binding buffer. Binding of CXCL10-AF647 to the cells was determined by flow cytometry (BD FACS Canto II) after addition of DAPI (1.25 µg/ml final concentration) to exclude dead cells. Competition binding assays were performed at 4 °C by incubating non-labeled chemokine shortly before adding fluorescently labelled CXCL10-AF647.

For quantification of binding affinity, curve fitting and $K_d$ values were determined using one site specific binding equation in GraphPad Prism 8 after subtracting total binding fluorescence values by the nonspecific fluorescence binding in the parental cell line, which does not express GPR182. In competitive binding experiments, curve fitting and $K_i$ values were determined using one site fit $K_i$ equation in GraphPad Prism 8 by providing the concentration (50 nM) and $K_d$ values of fluorescently labeled CXCL10-AF647 chemokine.

Unlabeled human chemokines were from Peprotech: CCL1 (300-37), CCL2 (300-04), CCL3 (300-08), CCL4 (300-09), CCL5 (300-06), CCL7 (300-17), CCL8 (300-15), CCL11 (300-21), CCL13 (300-24), CCL14 (300-38B), CCL15 (300-43), CCL16 (300-44), CCL17 (300-30), CCL18 (300-34), CCL19 (300-29B), CCL20 (300-29A), CCL21 (300-35A), CCL22 (300-36A), CCL23 (300-29), CCL24 (300-33), CCL25 (300-45), CCL26 (300-48), CCL27 (300-54), CCL28 (300-57), CX3CL1 (300-31), CXCL1 (300-11), XCL1 (300-20), CXCL2 (300-39), CXCL3 (300-40), CXCL4 (300-16), CXCL5 (300-22B), CXCL6 (300-

41), CXCL7 (300-14), CXCL8 (200-08), CXCL9 (300-26), CXCL10 (300-12), CXCL11 (300-46), CXCL12α (300-28A), CXCL12β (300-28b), CXCL13 (300-47), CXCL14 (300-50), CXCL16 (300-55). Unlabeled mouse chemokines were from Biolegend: CCL1 (584802), CCL25 (589302), CX3CL1 (583502), XCL1 (783502), CXCL3 (590802), CXCL5 (573302), CXCL7 (586702), or Peprotech: CCL2 (250-10), CCL3 (250-09), CCL4 (250-32), CCL5 (250-07), CCL6 (250-06), CCL7 (250-08), CCL8 (250-14), CCL9/10 (250-12), CCL11 (250-01), CCL12 (250-04), CCL17 (250-43), CCL19 (250-27B), CCL20 (250-27), CCL21 (250-13), CCL22 (250-23), CCL24 (250-22), CCL27 (250-26), CCL28 (250-30), CXCL1 (250-11), CXCL2 (250-15), CXCL4 (250-39), CXCL6_92aa (250-17), CXCL6_70aa (250-36), CXCL9 (250-18), CXCL10 (250-16), CXCL11 (250-29), CXCL12a (250-20A), CXCL12b (250-20B), CXCL13 (250-24), CXCL14 (250-45), CXCL16 (250-28),

<u>Determination of chemokine levels</u>

Mice were backcrossed onto a C57BL/6J background at least 8 to 10 times, and experiments were performed with littermates as controls. Male animals (12−16 weeks of age) were used and were housed under a 12-h light–dark cycle with free access to food and water under specific pathogen-free conditions. After euthanasia, blood from wild-type or GPR182$^{-/-}$ mice (2) was collected into tubes coated with clot activator and allowed to clot for 30 min at room temperature. Serum was collected after centrifugation at 12,000 x g for 10 min at room temperature and was frozen at -80°C until analysis. Chemokine levels were determined using ELISA kits for mouse chemokines (Abcam: XCL1 (ab269540), CCL24 (ab100681), CCL28 (ab210578), CXCL11 (ab204519); Thermo Fisher Scientific: CCL11 (EMCCL11), CCL19 (EMCCL19), CCL25 (EMCCL25), CCL27A (EMCCL27A), CXCL9 (EMCXCL9); RnD systems: CCL22 (MCC220); Novus Bio: CXCL14 (NBP2-70015)), following manufacturer’s instructions.

Statistics

Statistical analysis was performed using GraphPad Prism software (version 8). Analysis between two groups was performed with the unpaired two-tailed Student's t-test.

Study approval

Maintenance of the animals and experimental procedures were in agreement with the German animal welfare legislation and were approved by the local animal welfare authorities and committees (Regierungspräsidium Darmstadt, Germany).

## Supplemental Figures

### Suppl. Fig. 1

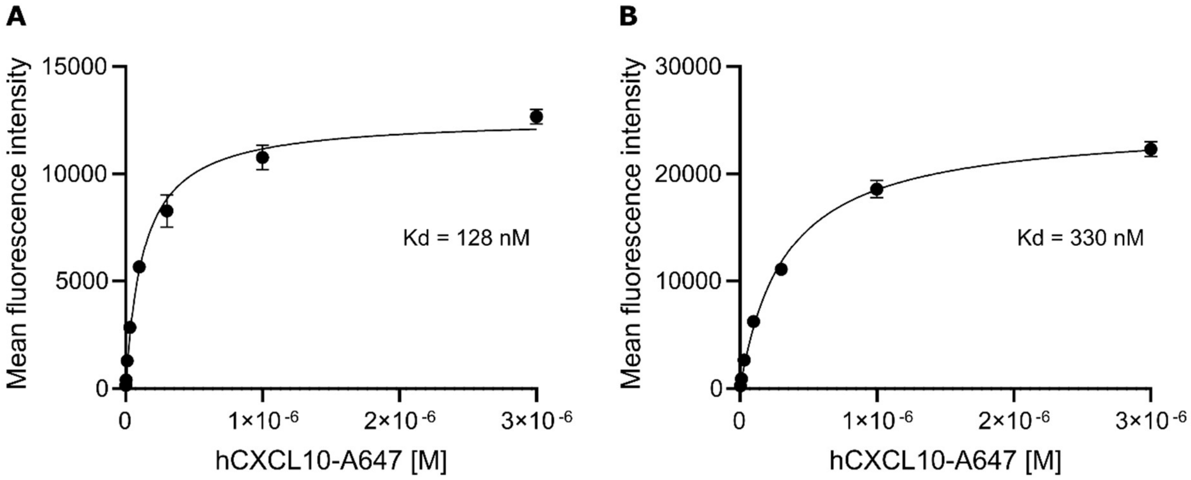


**Suppl. Figure 1.** Binding of the fluorescently labeled human chemokine CXCL12-AF647 to human (A) or mouse (B) GPR182 stably expressed by HEK-293 cells (n=3 biological replicates per concentration; shown is a representative of two independently performed experiments). Shown are mean values +/- S.D..

**Suppl. Fig. 2**

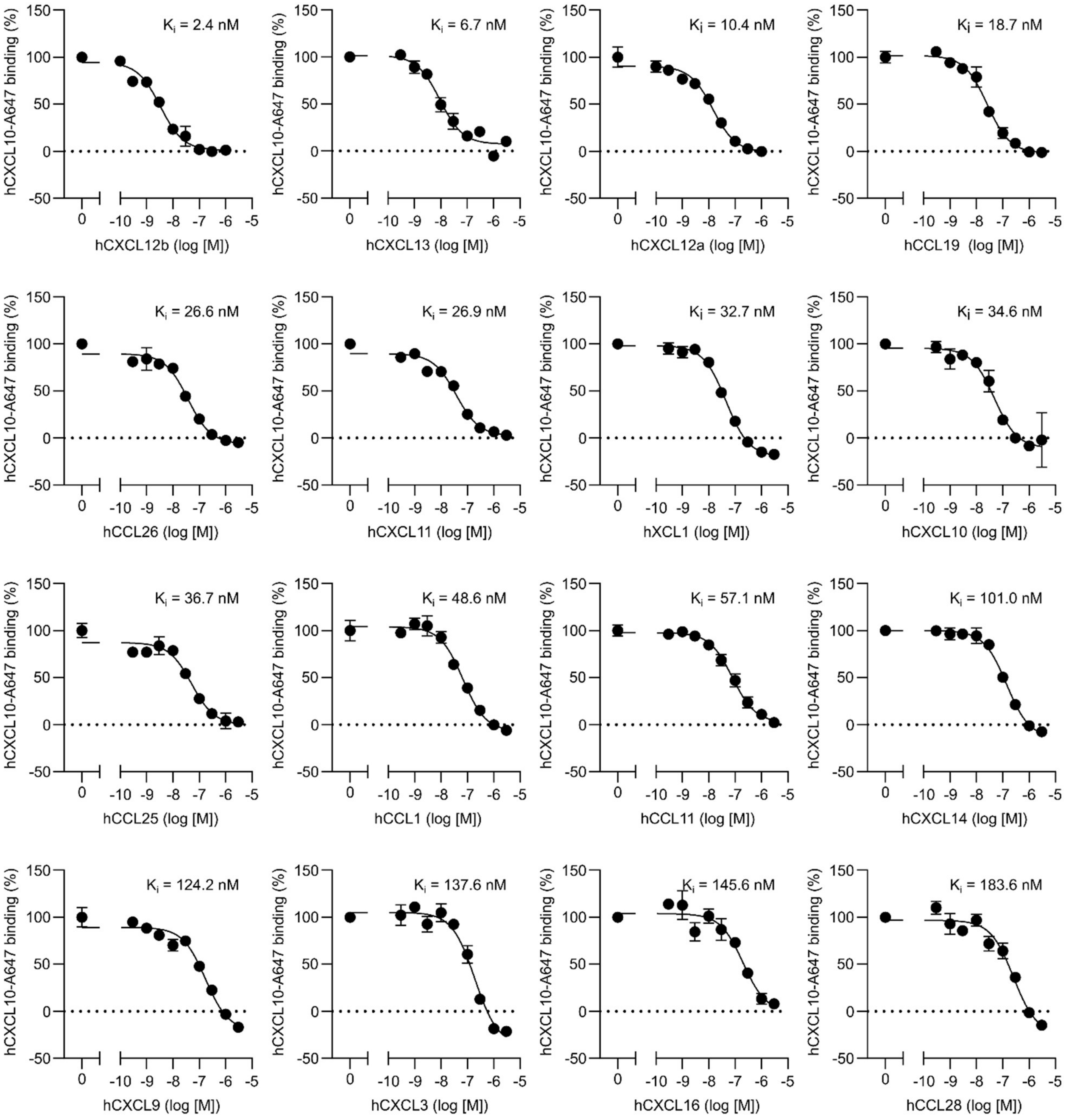


**Suppl. Figure 2.** Competition of hCXCL10-AF647 and the indicated unlabeled human chemokines for binding to human GPR182 expressed by HEK-293 cells (n=3 biologcial replicates per concentration; shown is a representative of two independently performed experiments). Shown are mean values +/- S.D..

**Suppl. Fig. 3**

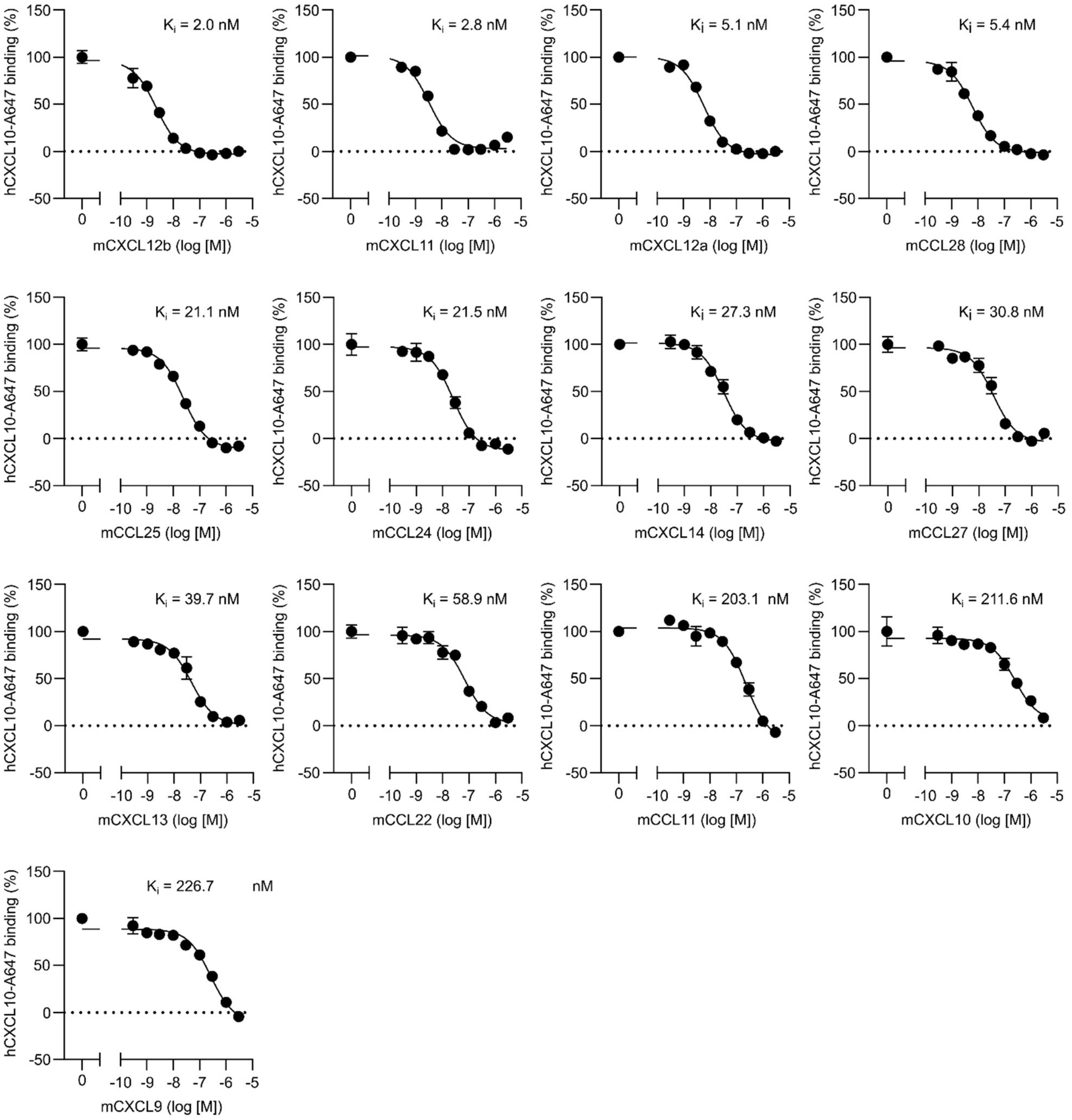


**Suppl. Figure 3.** Competition of hCXCL10-AF647 and the indicated unlabeled mouse chemokines for binding to mouse GPR182 expressed by HEK-293 cells (n=3 biological replicates per concentration; shown is a representative of two independently performed experiments). Shown are mean values +/- S.D..
